# Use of Valbenazine in a 54-Year-Old Female with Severe Tardive Dyskinesia

**DOI:** 10.7759/cureus.6801

**Published:** 2020-01-28

**Authors:** Maria Ruiza Yee, Eduardo D Espiridion, John Gurski

**Affiliations:** 1 Psychiatry, Reading Hospital Tower Health, West Reading, USA

**Keywords:** td, clozapine, valbenazine

## Abstract

Tardive dyskinesia (TD) is a serious and often irreversible involuntary muscle movement that involves the face, lips, tongue, trunk, and extremities. TD is a risk in the use of antipsychotic medications, whether it is typical or first generation or atypical or second-generation antipsychotic. The risk is highest in patients receiving long-term antipsychotic treatment. Before the availability of valbenazine, clozapine was used to reverse or at least ameliorate TD. We report a case of a patient on long-term antipsychotic treatment whose TD was initially reversed by clozapine but was completely reversed by valbenazine.

## Introduction

Antipsychotic medication is the cornerstone in the treatment of psychosis in schizophrenia, schizoaffective disorder, bipolar disorder as well as adjunctive therapy in major depressive disorder. The incidence of tardive dyskinesia (TD) in typical or first generation antipsychotic is 20-30% [1], while it is lower with atypical or second-generation antipsychotic at 13-15% [2]. While TD is a serious and often irreversible side effect of antipsychotic medication, discontinuation of antipsychotic medication is at times not possible as it results in worsening of the underlying psychiatric condition. Here, we describe a case of a woman who had long-term exposure to both typical and atypical antipsychotic due to the severity of her psychiatric illness, how discontinuation of the typical antipsychotic, and treatment with clozapine did not ameliorate the TD. But, use of valbenazine proved effective.

## Case presentation

A 54-year-old white female has a long history of schizoaffective disorder and intellectual disability dating back to 1987, when she was first hospitalized. She has had about 25 psychiatric hospitalizations since then. Some of her acute hospitalizations would last anywhere from three to five months. These acute hospitalizations occurred frequently. She was hospitalized almost monthly, when not hospitalized for a prolonged period of time. She was hospitalized at a state hospital for two years. She has attempted suicide five times: overdose, hanging with a rope, cutting her wrists. She was physically abused by biological mother, who was divorced from biological father when the patient was only a year old, and stepfather. Stepfather also sexually abused her. Because of physical and sexual abuse, the patient was placed in foster care from age 5-18. She struggled academically and was in special education classes. She never completed high school, dropping out after the 9th grade. She is single, having never married. She has no children. She was never employed. She is on permanent disability and lives in a personal care home. Throughout the years, the patient was treated with numerous antipsychotic medications including mesoridazine, trifluoperazine, haloperidol, risperidone, olanzapine, quetiapine, ziprasidone, aripiprazole, and clozapine. She started exhibiting the first signs of TD in 2008. It was mild and the involuntary muscle movement involved her tongue. At the time, she was on trifluoperazine. She was then started on clozapine. The trifluoperazine was continued. The TD movements resolved completely. However, by January 2017, TD movements resurfaced. TD movements involved the mouth, lips and tongue. Her speech was difficult to understand because of this. She was on haloperidol, benztropine, clonazepam and clozapine. By March 2018, her abnormal involuntary movement scale (AIMS) score was 25, even though she was only on clozapine. Other medications she was on included benztropine, amantadine, bupropion, mirtazapine and venlafaxine. Benztropine, amantadine and bupropion were discontinued as these were thought to exacerbate the TD. She was started on valbenazine, initially 40 mg daily and titrated to 80 mg daily two weeks later. One month later, patient’s TD movements were hardly noticeable. A year and a half later, AIMS score was two. Twenty months later, TD movements were completely resolved.

## Discussion

Antipsychotic medications remain the cornerstone in the treatment of psychosis. However, long-term treatment, which is inevitable in chronic conditions such as schizophrenia, schizoaffective disorder, and bipolar disorder, run the risk of TD. The risk of TD varies. The risk for first generation antipsychotic medication is higher than in second-generation antipsychotic medication (32.4% vs 13.1%) [1]. The most accepted hypothesis in its mechanism of action is prolonged blockade of postsynaptic dopamine receptors, leading to dopamine receptor supersensitivity, gamma-aminobutyric acid (GABA) depletion, cholinergic deficiency, oxidative stress, altered synaptic plasticity, neurotoxicity and defective neuroadaptive signaling [2]. Hence, it is advisable to consider the second-generation antipsychotic rather than the first generation antipsychotic when using antipsychotic medication. The Diagnostic and Statistical Manual of Mental Disorder, Fifth Edition (DSM-V) classifies TD as medication-induced movement disorder that can develop after short-term and long-term use of medications, as well as after discontinuation of, change in, or reduction in medications. In all cases, TD must persist for at least one month after a medication is discontinued for TD diagnosis [3]. Clozapine is the first atypical antipsychotic. Because of its adverse effect of agranulocytosis, it is not a first line antipsychotic medication. It is approved for use in refractory schizophrenia cases and in schizophrenia and schizoaffective disorder cases with recurrent suicidal behavior [4]. Prior reports show use of clozapine to reverse or ameliorate TD in patients exposed to long-term treatment with first generation antipsychotic medication [5, 6]. However, response to clozapine was variable, 40-60%. So, it was not surprising that our patient’s TD which initially resolved, resurfaced. Valbenazine seems to be a more effective treatment for TD. The mechanism of action is unknown but it is thought to be mediated through the reversible inhibition of vesicular monoamine transporter 2 (VMAT2), a transporter that regulates monoamine uptake from the cytoplasm to the synaptic vesicle for storage and release a vesicular monoamine transporter [7]. It is indicated for treatment of TD in adults. The efficacy of valbenazine was established in a six-week randomized, double-blind, placebo-controlled trial where patients with underlying schizophrenia, schizoaffective disorder and mood disorder had moderate to severe TD. Seventy percent of subjects were receiving atypical antipsychotics, 14% were receiving typical or combination antipsychotics, and 16% were not receiving antipsychotics. The primary efficacy endpoint was the mean change from baseline in the AIMS dyskinesia total score at the end of week 6. The change from baseline for two fixed doses of valbenazine (40 mg or 80 mg) was compared to placebo. At the end of week 6, subjects initially assigned to placebo were re-randomized to receive valbenazine 40 mg or 80 mg. Subjects originally randomized to valbenazine continued valbenazine at their randomized dose. Follow-up was continued through week 48 on the assigned drug, followed by a four-week period off-drug (subjects were not blind to withdrawal) [8].

## Conclusions

Antipsychotic medications are the cornerstone in the treatment of psychosis. However, long-term treatment risks the development of TD. Clozapine, the first atypical antipsychotic, was thought to be a possible treatment for TD. Unfortunately, response to clozapine was variable, 40-60%. Hence, the need for an alternative. Valbenazine seems to be an effective alternative.

## References

[1] Stegmayer K, Walther S, Van Harten P (2018). Tardive dyskinesia associated with atypical antipsychotics: prevalence, mechanisms and management strategies. CNS Drugs.

[2] Cornett E, Novitch M, Kaye AD, Kata V, Kaye AM (2017). Medication-induced tardive dyskinesia: a review and update. Ochsner J.

[3] American Psychiatric Association (2013). Diagnostic and Statistical Manual of Mental Disorders.

[4] Kane J, Honigfeld G, Singer J, Meltzer H (1988). Clozapine for the treatment-resistant schizophrenic: a double-blind comparison with chlorpromazine. Arch Gen Psychiatry.

[5] Dalack GW, Becks L, Meador-Woodruff JH (1998). Tardive dyskinesia, clozapine, and treatment response. Prog Neuropsychopharmacol Biol Psychiatry.

[6] Kumet R, Freeman MP (2002). Clozapine and tardive dyskinesia. J Clin Psychiatry.

[7] Correll CU, Kane JM, Citrome LL (2017). Epidemiology, prevention, and assessment of tardive dyskinesia and advances in treatment. J Clin Psychiatry.

[8] O'Brien CF, Jimenez R, Hauser RA, Factor SA, Burke J, Mandri D, Castro‐Gayol JC (2015). NBI-98854, a selective monoamine transport inhibitor for the treatment of tardive dyskinesia: a randomized, double-blind, placebo-controlled study. Mov Disord.

